# Occupational Exposure to Pesticides in Tobacco Fields: The Integrated Evaluation of Nutritional Intake and Susceptibility on Genomic and Epigenetic Instability

**DOI:** 10.1155/2018/7017423

**Published:** 2018-06-03

**Authors:** Vivian F. Silva Kahl, Varinderpal Dhillon, Michael Fenech, Melissa Rosa de Souza, Fabiane Nitzke da Silva, Norma Anair Possa Marroni, Emilene Arusievicz Nunes, Giselle Cerchiaro, Tatiana Pedron, Bruno Lemos Batista, Mónica Cappetta, Wilner Mártinez-López, Daniel Simon, Juliana da Silva

**Affiliations:** ^1^Laboratory of Toxicological Genetics, Post-Graduate Program in Cellular and Molecular Biology Applied to Health (PPGBioSaúde), Lutheran University of Brazil (ULBRA), Av. Farroupilha, 8001 Canoas, RS, Brazil; ^2^Health and Biosecurity Flagship, Commonwealth Scientific and Industrial Research Organization (CSIRO), Gate 13 Kintore Avenue, Adelaide, SA, Australia; ^3^Laboratory of Oxidative Stress, Post-Graduate Program in Cellular and Molecular Biology Applied to Health (PPGBioSaúde), Lutheran University of Brazil (ULBRA), Av. Farroupilha, 8001 Canoas, RS, Brazil; ^4^Laboratory of Experimental Hepatology-Physiology, Federal University of Rio Grande do Sul (UFRGS), Rua Ramiro Barcelos, 2350 Porto Alegre, RS, Brazil; ^5^Postgraduate Program in Biosystems, Foundation Federal University of ABC (UFABC), Av. dos Estados, 5001 Santo André, SP, Brazil; ^6^Postgraduate Program in Science and Technology/Chemistry, Foundation Federal University of ABC (UFABC), Av. dos Estados, 5001 Santo André, SP, Brazil; ^7^Laboratory of Genetic Epidemiology, Genetics Department, Medicine School, Universidad de la República, Gral. Flores, 2125 Montevideo, Uruguay; ^8^Epigenetics and Genomic Instability Laboratory, Instituto de Investigaciones Biológicas Clemente Estable, Montevideo, Uruguay; ^9^Laboratory of Human Molecular Genetics, Post-Graduate Program in Cellular and Molecular Biology Applied to Health (PPGBioSaúde), Lutheran University of Brazil (ULBRA), Av. Farroupilha, 8001 Canoas, RS, Brazil

## Abstract

Pesticides used at tobacco fields are associated with genomic instability, which is proposed to be sensitive to nutritional intake and may also induce epigenetic changes. We evaluated the effect of dietary intake and genetic susceptibility polymorphisms in *MTHFR* (rs1801133) and *TERT* (rs2736100) genes on genomic and epigenetic instability in tobacco farmers. Farmers, when compared to a nonexposed group, showed increased levels of different parameters of DNA damage (micronuclei, nucleoplasmic bridges, and nuclear buds), evaluated by cytokinesis-block micronucleus cytome assay. Telomere length (TL) measured by quantitative PCR was shorter in exposed individuals. Global DNA methylation was significantly decreased in tobacco farmers. The exposed group had lower dietary intake of fiber, but an increase in cholesterol; vitamins such as B_6_, B_12_, and C; *β*-carotene; and *α*-retinol. Several trace and ultratrace elements were found higher in farmers than in nonfarmers. The *MTHFR CT/TT* genotype influenced nucleoplasmic bridges, nuclear buds, and TL in the exposed group, whereas *TERT GT/TT* only affected micronucleus frequency. We observed a positive correlation of TL and lipids and an inverse correlation of TL and fibers. The present data suggest an important role of dietary intake and subjects' genetic susceptibility to xenobiotics-induced damages and epigenetic alterations in tobacco farmers occupationally exposed to mixtures of pesticides.

## 1. Introduction

Tobacco fields occupy 757,521 hectares in Brazil, and 95.4% of these are located in the South Region [1]. Tobacco leaf production plays an important role in the Brazilian economy, employing an enormous number of subjects in its entire production chain. This crop is farmed by families, involving whole communities in this stage of the productive chain [2]. It is therefore relevant that tobacco demands a great amount of pesticides to keep it free from pests [3]. More than 100 different pesticides are used worldwide for the production of tobacco, and the active ingredients of most pesticides are organic and/or inorganic. The latter in general include elements such as copper, sulfur, and potassium, while organophosphates are a class of pesticides more commonly used in agricultural areas.

Based on scientific evidence, it is fully justified to investigate the real, predicted, and perceived risks of pesticides endured by humans. Pesticides used at tobacco fields have been shown to increase DNA damage, as observed in cell lines [4–6], animal models [7, 8], and human studies [9–11]. They were also already evaluated either as single chemicals [6, 7, 12] or as mixtures (reviewed by [13]). The latter is the standard method of pesticide application in crop farming worldwide [13], including for Brazilian tobacco farmers [14–17]. Sensitive methods such as Comet assay [9, 18, 19], cytokinesis-block micronucleus (CBMN) assay [13–15, 20], and buccal micronucleus (BMCyt) assay [18, 19, 21] have been successfully applied in identification of DNA damage as a result of occupational risk to pesticides.

Formulations of pesticides used at tobacco fields contain inorganic elements, solvents, and metals [3]. Farmers working at tobacco fields have been found to present higher concentrations of several inorganic elements and metals [14, 16]. One of the major mechanisms of metals in carcinogenesis is inhibition of DNA repair through generation of reactive oxygen species. Generation of oxidative stress can lead to a homeostatic imbalance between pro- and antioxidant factors, resulting in oxidative damages to several molecules, including DNA [22]. Genetic conditions, such as different polymorphic metabolizing genes, and acquired conditions are thought to modify trace elements and metal homeostasis [22, 23].

More recently, telomere length has been introduced as a new biomarker in occupational exposure evaluations, including pesticides (reviewed by [24]). Telomeres mark the end of chromosomes and play a critical role in genomic stability by preventing chromosome end fusions. Telomerase is a reverse transcriptase enzyme particular to telomeres, working to maintain telomere length by adding the DNA repeat TTAGGG in human chromosome ends. The core enzyme is composed by a protein component with reverse transcriptase activity (TERT) and a RNA component (TERC). Several studies indicate that *TERT* rs2736100 polymorphism, located at intron 2 of the *TERT* gene, may modify telomere homeostasis and is mostly related to different types of cancers (reviewed by [25]).

Genome stability is proposed to be sensitive to nutrient intake. It has been a few years since recommended dietary allowances based on DNA damage were first proposed [26], although they were not changed based on those evidences, so far. It is known that several micronutrients are involved in DNA synthesis and repair, prevention of DNA damage, and maintenance of DNA methylation [27]. More than that, the telomere maintenance *in vivo* has been linked to intake and/or restriction of different nutrients and foods ([24], reviewed by [28]). The accurate significance of these data is still unclear as they are results from single studies ([24], reviewed by [28]), but the separate association of telomere maintenance and nutritional intake with cancer is widely known and accepted [24].

Alterations in DNA methylation patterns have been associated with different health outcomes, carcinogenesis being one of the major aspects [29]. A bulk of investigations have identified several classes of pesticides that modify epigenetic marks [30], including persistent organic pollutants [31], glyphosate [9], paraquat, and arsenic, among others [30]. The most common genetic variant in the *MTHFR* gene affecting its function is the 677C>T polymorphism (rs1801133), being highly investigated as a potential modifier on folate status and DNA methylation [27–29]. The methylenetetrahydrofolate reductase (*MTHFR*) gene plays a key role in folate metabolism and in the methyl donor pathway. MTHFR enzyme activity determines the bioavailability of folate for synthesis of dTTP or methionine, in addition to exertion of a function in arsenic metabolism and toxicity. Genetic variations in this gene can lead to impaired function or inactivation of its enzyme [26].

In this study, we hypothesized if dietary intake and genetic susceptibility of MTHFR (*rs1801133*) and TERT (*rs2736100*) have an effect on genetic and epigenetic instability borne by tobacco farmers due to occupational exposure to pesticides.

## 2. Materials and Methods

### 2.1. Study Design

This study was approved by the National Ethics Committee for Research, CONEP, Brazil (CAAE 35639814.5.1001.5349), and written inform consent was obtained from each individual before the research began. Individuals from Santa Cruz do Sul (S 29°43′59^″^ W 52°24′52^″^) and Sobradinho (S 29°25′17^″^ W 53°01′43^″^) (Rio Grande do Sul state, southern Brazil) were sampled between June and September 2015. The study involved a total of 80 individuals: 40 nonexposed and 40 exposed (tobacco farmers) to pesticides. In each group, subjects were paired by age and gender. All participants were nonsmokers.

All farmers were regularly exposed to pesticides about three days per week, 6–8 hours per day, comprising ~360 hours from June to September. Work was done mainly in an open field, and pesticide application was done via costal pump. According to farmers' questionnaires from this study, the main classes of pesticides (main active ingredients) used by them are organophosphates (glyphosate-based), dithiocarbamates (mancozeb), inorganic compounds (magnesium aluminium phosphide), and copper oxide (copper). While copper oxide and dithiocarbamates act as fungicides, glyphosate-based pesticides are herbicides and magnesium aluminium phosphide works as insecticide. Additionally, farmers mentioned to make a bulk use of fertilizers.

The nonexposed group consisted of indoor office workers living in the same area of exposed individuals, but at least 15 km away from any tobacco field. Blood samples collected by venipuncture were transported to the laboratory at 4°C and processed within 24 h after collection. Blood samples were collected during the pesticide application period, from both groups. All subjects involved in the study completed an adapted version of a questionnaire as regard lifestyle, work habits, and demographic data [32], in addition to completing the “The Alcohol Use Disorders Identification” Test (AUDIT; [33]). Participants consuming 300 mL of alcoholic beverages per week were considered habitual drinkers [33]. Participants suffering from any chronic disease and under 18 years were not included in the research.

### 2.2. Nutritional Data

Based on Fenech et al. [34], all participants were asked to fill in a food frequency questionnaire record intake of every meal, during a normal day in their routine, using food pyramid as reference. Average daily consumption was based on individuals' report of food frequency intake. The record intake was used to evaluate subjects' daily intake of kilocalories, carbohydrates (%), proteins (%), lipids (%), cholesterol (mg), dietary fibers (g), and saturated fat (g, %), as well as micronutrients [vitamin B_2_ (mg), vitamin B_3_ (mg), vitamin B_5_ (mg), vitamin B_6_ (mg), vitamin B_8_ (mg), folate (*μ*g), vitamin C (mg), vitamin E (mg), *β*-carotene (mg), and *α*-retinol (*μ*g)]. This data was obtained according to the software “Diet Win Professional” (Windows), recommended by the Brazilian Association of Nutrition (ASBRAN), which uses “Food Guide for the Brazilian population,” and the Institute of Medicine on Healthy Eating Index [35]. In addition, subjects' height and weight were obtained to calculate their body mass index (BMI), using the World Health Organization Food and Agricultural Organization [36] guideline.

### 2.3. Vitamin B_12_ Dosage

Vitamin B_12_ was dosed on each individual's serum by electrochemiluminescence by competition in a Cobas e601 equipment (Roche Diagnostics, Indianapolis, USA).

### 2.4. Trace and Ultratrace Element Dosage

Samples were analyzed by an inductively coupled plasma mass spectrometer (ICP-MS Agilent 7900, Hachioji, Japan). Prior to the analysis, 1 mL of blood was placed in a 15 mL tube and freeze-dried (L101, Liobrás, São Carlos, Brazil). Approximately 400 *μ*L of double-distilled HNO_3_ (Synth, Brazil) was added in each 15 mL tube. The tubes were heated during 3 h at 90°C in a digester block (Analab, Bischheim, France). After cooling, the volume was made up to 14 mL with deionized water (Milli-Q Millipore, USA) and injected in the ICP-MS. Blanks were analyzed in each batch. Certified reference materials for whole blood (Seronorm Trace Elements Blood L-2, ALS Scandinavia AB, Lulea, Sweden, and CRM-Agro caprine-blood low and high, USP, São Paulo, Brazil) and animal tissue (TORT-3, National Research Council Canada, Ontario, Canada) were run for method accuracy.

The monitored isotopes were ^27^Al, ^121^Sb, ^75^As, ^138^Ba, ^9^Be, ^209^Bi, ^111^Cd, ^43^Ca, ^52^Cr, ^59^Co, ^63^Cu, ^56^Fe, ^208^Pb, ^7^Li, ^24^Mg, ^55^Mn, ^202^Hg, ^95^Mo, ^60^Ni, ^39^K, ^78^Se, ^107^Ag, ^23^Na, ^51^V, and ^68^Zn. The found detection limits were 0.0725, 0.0002, 0.0201, 0.0230, 0.0, 0.0115, 0.0010, 27.32, 0.0060, 0.0037, 0.0281, 0.1465, 0.0003, 0.00825, 0.227, 0.00777, 0.0012, 0.0080, 0.04067, 0.8844, 0.0788, 0.0050, 18.68, 0.0088, and 0.1027 ng·mL^−1^, respectively. Instrumental conditions were previously described by Pedron et al. [37]. All the values found by ICP-MS are presented as ppb (ng/mL of blood) for ultratrace levels and ppm (*μ*g/mL) to trace levels.

### 2.5. Cytokinesis-Block Micronucleus (CBMN) Assay

The CBMN assay was performed accordingly to Fenech [38], with slight modifications. Cultures of whole blood in duplicate (0.5 mL each) were set up. Culture medium was composed by RPMI-1640 medium (Gibco, Billings, USA) containing 10% of fetal bovine serum (Gibco), 2 mM L-glutamine (Gibco), and 1 mM sodium pyruvate (Invitrogen). Phytohemagglutinin (Invitrogen, Carlsbad, USA) was added at 202 *μ*g/mL to stimulate cell division at time 0 h. Cultures were incubated at 37°C and 5% of CO_2_ in a humidified incubator. Cytochalasin B (Sigma, St. Louis, USA) was added at 6 *μ*g/mL after 40 h of culture, and cells were harvested 24 h later by density centrifugation with Ficoll (GE, Chicago, USA). Cells were transferred to slides by cytocentrifugation, fixed, and stained using Panoptic staining (New Prov, Pinhais, BR). Slides were labeled and scored blindly. From each subject, 400 cells were evaluated for mono-, bi-, multinuclear cells, apoptosis, and necrosis frequencies. Subsequently, 1000 binucleated (BN) cells were scored to determine the frequency of BN cells with one or more micronucleus (MN), nuclear plasmatic bridges (NPB), and nuclear buds (NBUD), to evaluate genome damage [38].

### 2.6. Telomere Length Assay

Telomere length was measured by quantitative real-time PCR assay [39, 40]. Firstly, genomic DNA was extracted from isolated lymphocytes using PureLink Genomic DNA isolation kit (Invitrogen, Waltham, USA) and then quantified by a NanoDrop 1000 spectrophotometer (Thermo Fisher Scientific, Scoresby, Australia). Each sample was then diluted as per experimental requirement (5 ng/*μ*L). Each qPCR reaction was performed in triplicate using both telomere and *36B4*-specific primers in a 96-well plate using the ABI 7300 Real-Time PCR Detection System (Life Technologies, Carlsbad, USA). In each run, a reference DNA sample isolated from the 1301 cell line was also included. The final concentrations of PCR reagents were as follows: 1x SYBR Green Master Mix (Life Technologies), 20 ng DNA, 0.2 *μ*mol of each telomere-specific primers (F: 5′-GGTTTTTGAGGGTGAGGGTGAGGGTGAGGGTGAGGGT-3′; R: 5′-TCCCGACTATCCCTATCCCTATCCCTATCCCTATCCCTA-3′), and *36B4* single copy gene primers (F: 5′-CAGCAAGTGGGAAGGTGTAATCC-3′; R: 5′-CCCATTCTATCATCAACG GGTACAA-3′). Amplification conditions were the same for both telomere and *36B4* runs: 10 min at 95°C, followed by 40 cycles of 15 sec at 95°C, and 1 min at 60°C, followed by dissociation stage. The ratio of the telomere (T) repeat copy number to the single-copy gene (S) was determined for each sample. A standard curve with a high correlation factor (*R*^2^ > 0.96) was required to accept the results from plates. Results were expressed as base pairs (bp).

### 2.7. Global DNA Methylation Assay

Global DNA methylation assay was performed as previously described [41]. Each sample was analyzed in duplicate. The percentage (%) of global DNA methylation was measured in DNA isolated from lymphocytes employing relative quantification of 5-methyl-2′-deoxycytidine (5-mdC) using liquid chromatography by HPLC. Genomic DNA (1 *μ*g) from each subject involved in study was denatured for 10 min at 94°C and cooled quickly for 5 min. Subsequently, samples were hydrolyzed with nuclease P1 and alkaline phosphatase to create 2′-de-oxymononucleosides, which were then column separated by HPLC dC18 Atlantis (Waters, Milford, USA) of reverse phase (2.1 × 20 mm). A mixture of the nucleosides deoxyadenosine (dA), deoxythymidine (dT), deoxyguanosine (dG), deoxycytidine (dC), 5-methyl-deoxycytidine (5mdC), and deoxyuridine (dG) was used as standard. Results were calculated by integration of the 5mdC peak area relative to global cytidine (methylated or not). Samples showing either a difference of more than 3% in 5mdC content or having low HPLC resolution were removed from the analysis (*n* = 3).

### 2.8. Genotyping Assays

*MTHFR* (rs1801133) and *TERT* (rs2736100) were genotyped by qPCR using SNP genotyping (TaqMan®) assays [Assay ID C_1202883_20 and C_1844009_10 for MTHFR and TERT, respectively (Applied Biosystems, Carlsbad, CA, USA)] according to the manufacturer's instructions in a ABI 7300 Real-Time PCR Detection System (Life Technologies, Carlsbad, USA).

### 2.9. Statistical Analysis

The Kolmogorov-Smirnov test was used to test the normality of variables. Analysis of variance (ANOVA) and Student's *t*-test were used to evaluate the variability within and between categories of nonexposed and exposed and to compare results among variables. Fisher's exact test was performed to analyze the frequencies of alleles in genotyping and categories within categorical variables between groups. The chi-square test was used to evaluate Hardy-Weinberg equilibrium and significance of differences in genotype frequency. Spearman's correlation (with Bonferroni correction) was performed to assess the relationship among variables for nonexposed and exposed groups. Differences were considered significant at *p* < 0.05. GraphPad Prism version 5.01 (GraphPad Inc., San Diego, CA, USA) and SPSS Inc. version 23.0 (IBM Corp., Armonk, NY, USA) were used for statistical analysis.

## 3. Results

This study evaluated 80 individuals, divided in two groups: nonexposed and exposed to pesticides. Each group was composed of 40 individuals, including 19 males and 21 females (Table 1). The average age of individuals of the nonexposed group was 45.6 (±1.7; standard error (SE)) years, while that for the exposed subjects was 45.0 (±1.8) years. There was no significant difference of gender frequency between groups, as much as the average age of males and females within and between each group (Table 1).

Exposed subjects declare to work since youth, with an average of 28.3 (±2.1) years working in tobacco fields, with no significant difference between males and females in this aspect (Table 1). As regard use of protective personal equipment (PPE), 32.5% of farmers enrolled in this study declared not to use it all, while 25.0% use only gloves. On the other hand, only 10.0% make use of complete PPE, which includes overall, boots, gloves, mask, hat, and goggles.

Individuals' nutritional intake data were obtained from nonexposed and exposed groups, based on individuals' reminder intake reports (Table 1). Fat intake (cholesterol) was significantly higher in the exposed group (*p* = 0.003; unpaired *t*-test). Among the investigated micronutrients, farmers also had significantly elevated dosages of vitamin B_6_ (*p* = 0.007), vitamin C (*p* = 0.041), and *β*-carotene (*p* = 0.006). Additionally, serum vitamin B_12_ level was also measured and found significantly elevated in the exposed group as well (*p* = 0.0004). There was no significant difference between nonexposed and exposed groups for BMI data, neither for males and females (Table 1).

CBMN assay was used to investigate DNA damage (Table 2). The exposed group had significantly higher frequencies of MN (*p* < 0.001), NPB (*p* < 0.001), NBUD (*p* < 0.001), and binucleated cells (*p* < 0.001), when compared to the nonexposed group. Furthermore, exposed individuals had significantly shorter telomeres (*p* = 0.015) and decreased level of global DNA methylation (*p* = 0.006) in comparison with nonexposed subjects (Table 2). For all parameters in Table 2, there was no significant difference between males and females within each group (data not shown).

Table 3 shows the blood concentration of trace and ultratrace elements, evaluated through ICP-MS. Exposed subjects had significantly increased concentrations of aluminum (*p* = 0.047), arsenic (*p* = 0.006), chromium (*p* = 0.027), copper (*p* = 0.003), nickel (*p* = 0.008), potassium (*p* = 0.037), and zinc (*p* = 0.030), in relation to nonexposed individuals. We also examined a possible correlation between the concentrations of Al, As, Cr, Cu, Ni, K, and Z, with years of exposure for tobacco farmers, and we found no significant correlation.

The genotype frequency data of the studied polymorphisms for the nonexposed and exposed groups are shown in Table 4. There was no deviation from Hardy-Weinberg expectations. The allele and genotype frequencies of the *MTHFR* gene were significantly different between nonexposed and exposed groups (*p* = 0.006 and *p* = 0.004, resp.). There was no statistical difference between the two groups for the *TERT* polymorphism frequencies (Table 4).

Spearman's test (with Bonferroni correction) was used to examine if there was any correlation between parameters in this study. NPBs were significantly correlated with MN (*r*_s_ = 0.391; *p* = 0.002) and NBUD with BMI (*r*_s_ = 0.373; *p* = 0.001). In the exposed group, there was a positive correlation of NBUD with MN (*r*_s_ = 0.470; *p* = 0.001). Also for farmers, telomere length was positively correlated with lipids (*r*_s_ = 0.385; *p* = 0.002) and inversely correlated with fibers (*r*_s_ = −0.364; *p* < 0.001). Although not statistically significant, the older the individuals are in the exposed group, and they had higher frequencies of NBUD (*r*_s_ = 0.290; *p* = 0.074).

We assessed the effects of *MTHFR* and *TERT* genotypes in different parameters of this study (Table 5). Individuals from the exposed group who carried the *MTHFR CT/TT* genotype showed significantly less NPB (*p* = 0.034) and NBUD (*p* = 0.013) frequencies, in addition to higher levels of DNA global methylation (*p* = 0.049; Table 5). For the nonexposed group, these same genotypes were associated with decreased frequencies of BN cells (*p* = 0.046; Table 5). Farmers with the *GT/TT* genotype in *TERT* showed significantly lower frequencies of MN (*p* = 0.029; Table 5) when compared to the *TERT GG* genotype. *TERT GT/TT* genotypes also showed associations in the nonexposed group by significantly reduced telomere length (*p* = 0.048; Table 5).

## 4. Discussion

Pesticides are complex mixtures and present genotoxic agents, and several studies have strongly associated occupational exposure with DNA damage [10, 14–16, 18, 19], oxidative stress generation [10, 14, 42], impairments to reproductive [42] and immune systems [43], and epigenetic alterations (reviewed by [25]), among others. Recently, a consortium called Agricultural Health Study (AHS) has approached the influence of exposure to pesticides in crop farms to telomere length, although those studies are not clear which pesticides, whether as pure or mixture use, and which crop are being investigated [44, 45]. Our group demonstrated shorter telomere length in tobacco farmers, exposed to both pesticides and nicotine from tobacco leaves, is associated with increased oxidative stress levels and trace and ultratrace element status [17, 46]. Along with the available data in literature, the influence of pesticides on telomere length demonstrates that occupational exposure to pesticides presents a long-term effect on farmers [7, 11, 13, 21, 30, 31, 42, 44, 47]. The wild type of *PON1 Gln192Arg*, a polymorphism directly involved in metabolizing organophosphate pesticides, was found to be associated with an increase in buccal micronucleus in tobacco farmers [16]. A significantly increased frequency of cell death in buccal cells was observed in tobacco farmers in all periods of the crop season [48].

A systematic review explored the influence of some classes of pesticides on cellular metabolism of lipids, proteins, and carbohydrates [49]. Their results indicated that organophosphates and carbamates impair the enzymatic pathways involved in metabolism of carbohydrates, fats, and proteins [49]. A study analyzed the influence of diet on the level of oxidative DNA damage and incorporated uracil and DNA repair capability in two groups of individuals with antagonist diets: the first group had a diet rich in organic products, whole grain, fruits, and vegetables, while the diet of the second group was rich in processed foods [50]. Results showed no differences as regard levels of misincorporated uracil or DNA repair ability. As expected, the group with a healthier diet (more fruits, vegetables, and no industrialized food) presented higher levels of vitamins A, B_6_, B_12_, and C and folic acid. This same group presented lower levels of oxidized purines and pyrimidines [50]. Tobacco farmers also showed increased levels of vitamins B_6_ and C and *β*-carotene on record nutritional intake data and of serum vitamin B_12_ level. However, increased genomic instability and epigenetic alterations were also observed in the exposed group, evidencing that this diet, although rich in micronutrients, was not sufficient to counterbalance the damage. Nascimento et al. [51] analyzed vitamin C content in children living in a tobacco farming area in addition to investigating health effects of environmental exposure to pesticides. They found increased oxidative damage and still higher levels of vitamin C, during blood sampling, in children whose parents are tobacco farmers. Authors suggest that, despite the elevated oxidative stress, children had also a higher defense against oxidative damage, mainly explained by an increased consumption of fruits and vegetables rich in vitamin C, such as orange and tangerine [51].

The CBMN assay is a reliable method in the investigation of cytogenetic damage in individuals exposed to several chemicals, in addition to investigating diet intake influence on genomic stability [52]. In the currently study, farmers exposed to mixtures of pesticides in tobacco fields for an average of 28 years had significantly increased frequencies of MN, NPBs, NBUD, and binucleated cells. Although there are different findings as regard crops, formulation of pesticide mixtures, and schedule of crop treatments, among others, most studies that evaluate pesticide sprayers found an increase in MN frequencies [13]. Spraying increases the different routes of pesticide absorption, which may occur via cutaneous, respiratory, and oral routes mainly [42]. About 90% of tobacco farmers involved in this work did not use PPE properly, which makes the occurrence of different genotoxic damages highly likely. A recent study with tobacco farmers showed that the use of at least part of PPE protected farmers from an increase in MN frequency [14]. The increased risk of Parkinson's disease (PD) has been widely linked to pesticide exposure through many epidemiological studies (reviewed by [42]). The AHS, in association with other prospective studies, observed that use of gloves and hygiene at the workplace can reduce significantly the association of pesticide exposure and PD [53]. However, Furlong et al. [53] were able to show that use of protective gloves demonstrated no association of PD with pesticide permethrin and paraquat occupational exposure.

Telomere length has become a prospective biomarker of occupational exposure. Duration of work exposed to welding fumes was associated with longer telomeres in one study [54, 55], besides an interaction with LINE-1 and *Alu* methylation levels, reflecting the epigenetic pathway involved. Workers of fitness equipment manufacturing had shorter telomeres than office workers from the same industry, in addition to increased levels of Cr and Mn [56]. The occupational exposure to coal tar pitch leads to a decrease in TL in coal miners; however, no methylation of promoter genes was found [57]. Ziegler et al. [11] showed accelerated telomere shortening in blood leukocytes after occupational exposure to biphenyls, while Bassig et al. [58] reported an opposite result in individuals occupationally exposed to benzene. In the case of occupational exposure to pesticides, most of the studies arise from the AHS consortium, evidencing, in the majority, telomere reduction [45]. A recent work demonstrates that children environmentally exposed to pesticides at the same tobacco-producing region of samples from our study had increased oxidative damages, in addition to increased levels of Cr associated with alterations in hematological parameters [51]. Our current results show, beyond significantly shorter telomere length in tobacco farmers occupationally exposed to mixtures of pesticides, global DNA hypomethylation. Another recent study from our group proposed, by a systems biology approach, a mechanism by which pesticides used in tobacco farming may decrease telomere length. Through an epigenetic process, pesticides may block AKT1 protein, decreasing ubiquitin-proteasome system activity, which increases levels of TRF1 and TERT. TRF1 is a major component of the shelterin complex. In other words, while TERT promotes telomerase activity, the shelterin complex becomes tighter, ultimately blocking telomerase activity [47].

There is increasing evidence that nutritional factors are associated with telomere length (reviewed by [22]), although these findings are still under investigation. Folate and vitamin B_12_ play an important role in the maintenance of nuclear integrity. Their deficiency was related, until recently, to decreased telomere length [26]. In our study, there was no significant difference in folate between nonexposed and exposed groups; still, vitamin B_12_, measured in serum, was significantly elevated for farmers, and yet, a decrease in telomere length was found for the same group. In other study, folate deficiency leads to long but dysfunctional telomeres, associated with higher chromosome instability, probably due to DNA hypomethylation (reviewed by [24]). Paul et al. [59] observed that levels of folate above the median have a positive correlation with telomere length. Authors suggest that folate influences telomere by modifying DNA integrity and by exerting an epigenetic regulation on telomere length through DNA methylation [59]. The adequate intakes of folate and vitamin B_12_ were associated with increased genomic instability in tobacco farmers when compared to the ones with inappropriate intake of those micronutrients [60]. Our study differs from this one because participants from the current work, whether exposed or not, had appropriate intake of folate and B_12_. This adds to growing evidence that, actually, there is a complex relationship of telomere length with B vitamins (reviewed by [24]). In the general US population exposed to cadmium, leukocyte telomere length was found to be decreased and a diet pattern was also evaluated. Similarly, to our data, Nomura et al. [61] did not find any association of vitamin B_12_ with TL, and regardless of which vitamins and carotenoids were evaluated, their concentrations did not modify blood cadmium and leukocyte TL associations [61]. The high complexity of mixtures of pesticides may also impose a quite different challenge for DNA repair and integrity maintenance, even if individuals have a proper diet.

In our study, we observed a positive correlation between telomere length and intake of lipids and an inverted correlation with fiber intake. Fiber is a dietary factor that can reduce lipids levels through absorption of fat (reviewed by [24]). A high-fat diet intake was inversely associated with telomere length in more than 2000 American women, and individuals making use of vitamin complexes may have telomeres ~273 bp longer than those not using (reviewed by [24]). Human diet has a multifactorial nature: the relation between nutrition, xenobiotics exposure, and genetic structure is complex and can affect similar biological pathways. Besides, interindividual differences in activating and detoxifying substances are crucial for human health.

A recent study reported the association between lower levels of global DNA methylation and shorter telomere length in healthy adolescents, mainly linked to ethnicity [62]. Risk factors for lower DNA global methylation include environmental factors, such as exposure to persistent organic pollutants, benzene, and arsenic [29]. A longitudinal study of boilermakers found significant positive associations between global DNA hypomethylation and decreased telomere length in peripheral blood leukocytes [54]. In the current analysis, we did not find a correlation between telomere length and DNA methylation, but both biomarkers were found significantly altered in the exposed group. However, when farmers were genotyped for the *MTHFR* gene, exposed individuals with the minor allele (*CT* or *TT*) showed hypermethylation in relation to the *CC* ones. Interestingly, the polymorphic genotype increased DNA methylation in farmers, in addition to folate as well, although not significantly (data not shown). An intervention study showed an increase in global DNA methylation after 7 weeks of supplementation with folate only for individuals with the polymorphic *MTHFR C677T* genotype (reviewed by [29]). Another pilot intervention trial induced methylation changes in genes involved in mitochondrial oxidative metabolism, by exposing individuals to particulate matter (PM_2.5_). B-vitamin supplementation, containing B_6_, folate, and B_12_, avoid those changes [63]. This outcome was linked to inflammatory response after exposure, which is highly possible as DNA methylation plays a role on protein biomarkers of the immune system, as analyzed in over 10 million SNPs [64].

The *CC* polymorphism of *MTHFR* was adverse in tobacco farmers, promoting an increase in NBUD and NPB in relation to the *CT/TT* polymorphism. NBUD were 27% lower in *MTHFR TT* human lymphocytes than in *MTHFR CC* [65]. Folate deficiency was shown to induce MN, NPB, and NBUD. There was no significant difference in folate levels between nonexposed and exposed groups in our study. Regardless of folate levels, our data is coherent with findings in literature in relation to a decrease in nuclear abnormalities, including MN frequencies, and the *MTHFR C677T* polymorphism: in the study of Kimura et al. [65], MN were found to be decreased in individuals carrying the *CC* genotype, and although it was not significant, the same was found in our exposed group.

Telomerase reverse transcriptase (TERT), the telomerase catalytic subunit, maintains telomere stability. The rs2736100 T>G polymorphism has been widely associated with a variety of neoplasia, mainly with shortened telomeres as a result, although the findings are yet inconclusive [66]. The micronucleus has been reported as an important biomarker in carcinogenesis [52]. In the current study, farmers harboring the *TERT GG* genotype had nearly 1.5-fold increased levels of MN when compared to farmers who were harboring the *TERT GT/TT* genotype. On the other hand, in the exposed group, the *GG* genotype did not influence telomere length, showing neither favorable nor unfavorable role. Different studies have confirmed that rs2736100 T>G polymorphism confers increased overall cancer risk [25, 66]. The *GG* genotype may upregulate *TERT* expression through which its oncogenic effect is exerted [66]. Interestingly, *TERT* mRNA expression was found to be significantly associated with arsenic (As) concentrations of drinking water and subjects' nails in a Chinese population exposed to this metal [67]. In addition to water and nail concentrations of As, Mo et al. [67] observed that pesticide use (mainly organochlorines and organophosphates) was significantly associated with *TERT* expression. MCF-7 cells treated with the organochlorine endosulfan showed increased levels of *TERT* mRNA expression [68], demonstrating that TERT may exert a role in pesticide exposure.

Although it can be found naturally, As is known as a water contaminant and as carcinogenic to humans, in addition to causing epigenetic modifications (reviewed by [69]). This metal was found significantly elevated in tobacco farmers in relation to the nonexposed group. Children whose parents are tobacco farmers were found to have increased levels of As as well, probably due to exposure to As following pesticide application, through inhalation of dusts [51]. Many tobacco farmers use As-based insecticides to avoid insect infection, such as glyphosate-based pesticides [69]. Repeated oxidative methylation and reduction reaction of As lead to a generation of methylated metabolites [70]. Altered DNA methylation is among the more plausible biological explanations for As-induced carcinogenesis. Hypomethylation may result from S-adenosyl methionine (SAM) depletion as it is required for both DNA methylation and inorganic As biomethylation [70].

Tobacco farmers during the pesticide application period also had significantly increased levels of aluminum (Al), chromium (Cr), copper (Cu), nickel (Ni), potassium (K), and zinc (Zn) in comparison with the nonexposed group. Two other studies with tobacco farmers observed increased levels of Al, Cr, K, and Zn [14, 16], notably because those elements are components of pesticides, including the ones used at tobacco crops [3]. It is worth noting that glyphosate-based pesticides can contain As, Cr, and Ni; dithiocarbamate may have Zn as a compound, while inorganic compounds, such as magnesium aluminum phosphide, contain K and Al. Nickel is known to be a human carcinogen, although the mechanism is largely unheard [70, 71]. It has been suggested that Ni carcinogenic potential relates to its ability to accelerate epigenetic changes including DNA methylation. On a former study from our group, the shorter TL in tobacco farmers was related to hypermethylation of the *p16* gene [46]. Nickel was found to promote gene-specific hypermethylation of the tumor suppressor p16 in several studies (reviewed by [70]).

Chromium was found increased in tobacco farmers in relation to the nonexposed group. Chromium has been reported to prompt a wide spectrum of DNA damage [71, 72], increasing the risk of neoplasm [72]. This metal was also found to promote gene-specific methylation on *p16* and on the 45S RNA gene [72]. Cr blood levels were above the recommended limits by the World Health Organization in samples from children living in tobacco farms, where their parents use pesticides with high Cr content, such as Orthene® and Talstar® [51]. A recent study showed that Cr content levels in tobacco farms are above or very close to the Brazilian limit established for agricultural use [3] and that glyphosate-based pesticides may contain Cr [69]. Chromium and copper (Cu) seem to be some of the most studied metals as regard human exposure. Copper is found in copper oxide fungicides used in tobacco fields [15]. Occupational exposure to Cu has shown also to result in different DNA damages (reviewed by [71]). Although methylation was still not related to human Cu occupational exposure, in addition to its genomic toxicity, Cu can alter histone acetylation and histone deacetylase activity [73]. Excessive exposure to Cu in a processing industry leads to increased DNA damage and several parameters of oxidative stress, along with alterations in pulmonary function parameters [74]. In fact, oxidative stress may facilitate tumorigenesis by dysregulating cellular antioxidants, disrupting cell growth and proliferation [75], which are enabled by telomere balance in length, structure, and proper function [76]. This disruption can be highly dependent on the duration of persistent exposure to carcinogenic elements [75], even when there is no dose-response in trace-element exposure. Such status is due to the metal-induced oxidative stress-potentiating manner contributing to carcinogenicity. Low doses of metals can induce tumor initiation while highly cytotoxic doses, such as found in our study, evoke free radicals [75].

In a recent review, high Se exposure showed to lead to inhibition of DNA methyltransferase expression and/or activity [77]. The evidence arises from studies in human cancer cell lines, rodents, and human studies cancer-free subjects [77]. The role of Se-induced DNA damage was shown to be oxygen-mediated, highlighting its oxidative stress pathway. This trace element was found significantly decreased in tobacco farmers from our study, although it is an essential micronutrient to humans. Supplementation with Se may protect DNA against formation of adducts, DNA or chromosome breakage (including one main mechanism of telomere fusion, called breakage-fusion-bridge cycle), and chromosome gain or loss. Sodium selenite was shown to improve cellular telomerase activity. Telomere length was extended after a 4-week treatment of Se in hepatocytes L-02 with a nutritional dose [78]. Selenium also affects methylation of human tumor-specific genes [77], modulation of global DNA methylation, and inhibition of histone deacetylation [79]. Different xenobiotic exposures may influence trace elements and metals' homeostasis, according to two different reviews [22, 23].

Our study has some limitations. Although subjects from both groups were paired by age and gender, the sample size is small. Additionally, dietary intake was assessed using a food frequency questionnaire. Only vitamin B_12_ and trace and ultratrace elements were quantified in biological samples. This highlights the need of future studies, mainly follow-up ones, that need to be undertaken with tobacco farmers. Knowledge of the genetic structure of populations is an important factor considering public health, including individual's nutrition, lifestyle, and environment. In this aspect, the current study sheds some light on the complex picture of occupational exposure to pesticides and its association with different biomarkers of DNA damage and the effects of nutritional intake and genetic susceptibility.

## 5. Conclusion

Altogether, results from this study highly link genomic instability, as shown by an increase in DNA damage in CBMN assay and by shorter telomere length, to the occupational exposure to mixtures of pesticides at tobacco fields. Our data also shows the role played by dietary intake and polymorphisms *MTHFR C677T* and *TERT* rs2736100 on the genomic instability of those farmers. It is already known that genome stability is sensitive to nutritional intake, particularly to those nutrients and foods that have a more significant effect on DNA synthesis and repair. Diet patterns may also play a role in telomere length dynamics. We have found an association of telomere length and micronutrients and with DNA hypomethylation in tobacco farmers. *TERT* and *MTHFR* genes were shown to have an influence on the frequency of nuclear aberrations and telomere length. The use of the genetic structure to understand the predisposition of subjects to xenobiotics-induced damages and epigenetics to comprehend expression is fundamental to providing a more refined recommendation of work safety guidelines for tobacco farmers. We suggest that further studies be undertaken with this population to enhance understanding of nutrigenomics and epigenetic effects on occupational exposure biomarkers.

## Figures and Tables

**Table 1:** Population descriptive, including nutritional intake data (based on individuals' reminder intake) and vitamin B12 serum level.

Parameters	Nonexposed (*n* = 40)	Exposed (*n* = 40)	*p* value^a^
Gender (*n*, %)			
Male	19 (47.5)	19 (47.5)	>0.999^b^
Female	21 (52.5)	21 (52.5)	
Age (mean ± SE)	45.6 ± 1.7	45.0 ± 1.8	0.797
Male	45.3 ± 2.6	44.5 ± 2.7	0.824
Female	45.8 ± 2.4	45.4 ± 2.5	0.891
*p*^c^	0.8907	0.8173	
Years of work in tobacco fields (mean ± SE)	—	28.3 ± 2.1	
Male	0	29.8 ± 3.2	
Female	0	26.8 ± 2.8	
*p*^c^	—	0.4919	
Body mass index (BMI)	27.8 ± 0.8	27.6 ± 0.9	0.844
Male	27.2 ± 0.8	25.5 ± 0.8	0.120
Female	27.1 ± 1.0	28.4 ± 1.2	0.439
*p*^c^	0.9360	0.1000	
Dietary intake			
Lipids (%)^d^	31.8 ± 1.2	30.4 ± 0.8	0.333
Cholesterol (mg)	247.1 ± 20.5	339.1 ± 22.0	**0.003**
Fibers (g)	26.5 ± 1.0	24.8 ± 1.4	0.312
Vitamin B_6_ (mg)	1.86 ± 0.07	2.24 ± 0.11	**0.006**
Folate (*μ*g)	177.5 ± 15.3	180.3 ± 13.0	0.890
Vitamin C (mg)	91.5 ± 11.3	124.2 ± 10.7	**0.041**
Vitamin E (mg)	34.8 ± 2.2	36.4 ± 2.8	0.666
B-carotene (mg)	474.2 ± 56.2	724.5 ± 66.1	**0.006**
A-retinol (*μ*g)	556.8 ± 52.5	473.7 ± 34.3	0.362
Vitamin B_12_ (pg/mL)	400.6 ± 18.2	564.0 ± 33.7	**0.0004**

^a^ *p* value between nonexposed and exposed groups; ^b^Fischer's exact test; ^c^*p* value between male and female individuals within each group; ^d^on a daily 2000 calorie diet. SE: standard error.

**Table 2:** CBMN parameters, telomere length (bp), and global DNA methylation (%) results for nonexposed and exposed groups. Data presented as mean ± standard error (SE).

Parameters	Nonexposed (*n* = 40)	Exposed (*n* = 40)	*p* value^c^
*CBMN*			
Micronucleus^a^	2.9 ± 0.4	6.0 ± 0.5	**<0.001**
Nucleoplasmic bridge^a^	2.6 ± 0.4	6.0 ± 0.5	**<0.001**
Nuclear buds^a^	2.0 ± 0.4	4.0 ± 0.4	**<0.001**
Binucleated cells^b^	61.3 ± 8.2	120.6 ± 8.4	**<0.001**
Apoptosis^b^	3.4 ± 0.4	3.9 ± 0.6	0.479
Necrosis^b^	3.6 ± 0.6	3.7 ± 0.8	0.978
*Telomere length (bp)*	4551 ± 145.5	4098 ± 105.3	**0.015**
*DNA global methylation (%)*	3.804 ± 0.206	3.023 ± 0.131	**0.006**

^a^Frequency per 1000 BN cells; ^b^frequency per 500 cells; ^c^*p* value between nonexposed and exposed groups; unpaired *t*-tests.

**Table 3:** Concentration of trace (*μ*g/mL) and ultratrace elements (ng/mL) determined in whole blood for both nonexposed and exposed groups. Data presented as mean ± standard error (SE). Unpaired *t*-test.

Element	Nonexposed (*n* = 40)	Exposed (*n* = 40)	*p* value
Aluminium (Al)	112.60 ± 12.00	230.50 ± 37.10	**0.046**
Antimony (Sb)	16.15 ± 2.64	19.17 ± 3.27	0.474
Arsenic (As)	3.61 ± 0.70	7.88 ± 1.27	**0.006**
Barium (Ba)	25.37 ± 3.30	19.62 ± 2.08	0.310
Beryllium (Be)	0.11 ± 0.01	0.09 ± 0.01	0.548
Bismuth (Bi)	0.83 ± 0.09	0.67 ± 0.09	0.211
Cadmium (Cd)	0.83 ± 0.07	0.99 ± 0.08	0.169
Calcium (Ca)	312,752 ± 34,868	271,559 ± 36,957	0.420
Chromium (Cr)	5.08 ± 0.66	8.44 ± 1.30	**0.026**
Cobalt (Co)	0.48 ± 0.06	0.59 ± 0.07	0.261
Copper (Cu)	3322 ± 267.60	4785 ± 380.90	**0.003**
Iron (Fe)^a^	2,580,203 ± 109,596	2,657,316 ± 110,552	0.622
Lead (Pb)	73.49 ± 8.48	97.88 ± 12.42	0.181
Lithium (Li)	0.17 ± 0.79	0.96 ± 0.90	0.513
Magnesium (Mg)^a^	158,025 ± 4678	149,515 ± 3298	0.145
Manganese (Mn)	74.92 ± 4.33	71.08 ± 2.71	0.651
Mercury (Hg)	2.74 ± 0.45	1.80 ± 0.22	0.251
Molybdenum (Mo)	1.95 ± 0.30	2.41 ± 0.28	0.269
Nickel (Ni)	2.75 ± 0.50	5.90 ± 0.85	**0.008**
Potassium (K)^a^	6,380,585 ± 728,826	8,300,853 ± 284,196	**0.038**
Selenium (Se)	762.50 ± 37.21	640.50 ± 23.27	**0.007**
Silver (Ag)	0.54 ± 0.07	0.47 ± 0.09	0.514
Sodium (Na)^a^	6,232,252 ± 737,640	5,730,624 ± 795,854	0.645
Vanadium (V)	20.48 ± 1.65	19.76 ± 1.63	0.758
Zinc (Zn)^a^	24,793 ± 1396	29,155 ± 970.60	**0.030**

^a^Trace elements.

**Table 4:** Genotype frequency distribution and allelic frequencies of *MTHFR* (rs1801133) and *TERT* (rs2736100) polymorphisms for nonexposed and exposed groups.

Genotype	Nonexposed (*n* = 40)	Exposed (*n* = 40)
MTHFR		
CC	17	10
CT	21	15
TT	2	13
Allelic frequencies		
C	0.69	0.46
T	0.31	0.54
TERT		
GG	12	7
GT	16	19
TT	12	13
Allelic frequencies		
G	0.50	0.42
T	0.50	0.58

**Table 5:** Micronucleus (MN), nucleoplasmic bridge (NPB), nuclear buds (NBUD), and binucleated (BN) cell frequencies, telomere length (bp), and % of global DNA methylation for nonexposed and exposed groups according to *MTHFR* and *TERT* polymorphisms. Data presented as mean ± standard error (SE).

Parameters	*MTHFR* (rs1801133)		*p* ^a^	*TERT* (rs2736100)		*p* ^b^
	CC (*n*)	CT/TT (*n*)		GG (*n*)	GT/TT (*n*)	
*CBMN*						
Micronucleus^c^						
Nonexposed	3.2 ± 0.7 (16)	2.6 ± 0.4 (23)	0.427	2.6 ± 0.5 (11)	2.9 ± 0.5 (28)	0.766
Exposed	5.0 ± 0.8 (10)	6.2 ± 0.6 (28)	0.307	7.6 ± 1.4 (7)	4.9 ± 0.5 (32)	**0.029**
Nucleoplasmic bridge^c^						
Nonexposed	2.2 ± 0.4 (17)	2.7 ± 0.7 (23)	0.555	2.2 ± 0.6 (11)	2.7 ± 0.5 (28)	0.589
Exposed	6.6 ± 0.7 (10)	4.7 ± 0.5 (28)	**0.034**	5.0 ± 1.0 (7)	5.5 ± 0.5 (32)	0.641
NBUD^c^						
Nonexposed	1.5 ± 0.4 (17)	2.2 ± 0.5 (23)	0.439	2.0 ± 0.5 (11)	2.0 ± 0.6 (28)	0.937
Exposed	4.7 ± 0.4 (10)	3.0 ± 0.3 (28)	**0.013**	3.6 ± 0.6 (7)	3.1 ± 0.3 (32)	0.535
Binucleated cells^d^						
Nonexposed	56.3 ± 7.0 (17)	38.1 ± 3.9 (20)	**0.046**	49.5 ± 7.1 (12)	44.9 ± 4.9 (28)	0.616
Exposed	106.7 ± 18.6 (10)	121.8 ± 9.6 (28)	0.441	135.3 ± 15.0 (7)	115.1 ± 9.7 (31)	0.359
Telomere length						
Nonexposed	4266 ± 150.7 (16)	5497 ± 468.4 (23)	0.053	5824 ± 713.9 (11)	4392 ± 125.1 (28)	**0.049**
Exposed	3918 ± 206.9 (10)	4406 ± 200.3 (28)	0.164	4504 ± 765.9 (7)	4161 ± 122.4 (31)	0.656
Global DNA methylation						
Nonexposed	3.779 ± 0.34 (16)	3.821 ± 0.26 (23)	0.922	3.659 ± 0.32 (10)	3.862 ± 0.26 (25)	0.662
Exposed	2.784 ± 0.24 (10)	3.335 ± 0.14 (28)	**0.049**	2.596 ± 0.26 (5)	3.075 ± 0.12 (30)	0.161

^a^ *p* value between the *CC* genotype and the combination *CT/TT* within groups; ^b^*p* value between the *GG* genotype and the combination *GT/TT* within groups; ^c^evaluated in 1000 BN cells per individual; ^d^evaluated in 500 cells per individual.
